# Study on roadway deformation and control method under large mining height conditions

**DOI:** 10.1038/s41598-026-38439-1

**Published:** 2026-04-15

**Authors:** Yuxiang Cao, Jiting Liu, Shuoyin Wang, Chong Zhang, Tao Song, Yunlong Cong, Haojie Xue, Jinkai Zhang, Yakun Huang, Haibin Jia

**Affiliations:** 1 Shenmu Nimgtiaota Coal Mining Company Ltd., Shaanxi Coal and Chemical Industry Group Company Ltd., Yulin, 719300 China; 2 School of Mechanics and Civil Engineering, China University of Mining and Technology (Beijing), Beijing, 100083 China; 3 State Key Laboratory for Tunnel Engineering, China University of Mining and Technology (Beijing), Beijing, 100083 China; 4 State Key Laboratory for Tunnel Engineering, Shandong University, Jinan, 250061 China; 5 School of Civil Engineering, Shandong University, Jinan, 250061 China; 6 China Coal Construction & Installation Engineering Group Co., Ltd., Handan, 056002 Hebei China; 7 Shandong New Dragon Energy Co., Ltd., Heze, 274918 Shandong China

**Keywords:** Large mining height, Gob-side entry retention, Soft mold fling wall, Mechanical model, Energy science and technology, Engineering, Solid Earth sciences

## Abstract

Focusing on the S1212 working face in Ningtiaota coal mine, this study investigates a soft mold filling wall as a primary support strategy for gob-side entry retention. The research aims to overcome the challenges of poor rock stability and complex support requirements under hard roof and large mining height conditions. A structural mechanical model of the surrounding rock was developed, from which a quantitative relationship between the support resistance of the soft mold filling wall and mining height was analytically derived. Numerical simulations were conducted to systematically investigate the effects of varying mining heights and wall widths on wall vertical stress, solid coal rib stress, and roadway deformation, thereby elucidating the load-bearing behavior of the wall and identifying optimal wall widths for different mining heights. The results demonstrate that: (1) At a constant mining height, vertical stress within the wall decreases progressively with increasing wall width, whereas at a constant wall width, increasing the mining height markedly amplifies vertical stress. (2) With increasing wall width, the solid coal rib peak stress decreases and its maximum shifts deeper into the rock mass, in contrast, under larger mining heights, the peak stress intensifies and migrates toward shallower zones. (3) Across varying mining heights, roadway floor heave exhibits an overall reduction with increasing wall width, however, the mitigation effect plateaus once the wall width surpasses a critical threshold. Integrating theoretical derivation with numerical analysis, the optimal wall width for the S1212 working face was determined as 1.4 m. Field validation further demonstrated that when the working face advanced 80 m, deformation of the surrounding rock stabilized, with a convergence deformation of 48.8 mm, thereby ensuring effective roadway stability control.

## Introduction

In traditional longwall mining, a coal pillar of specified width is usually preserved along the roadway to withstand mining-induced pressure resulting from the collapse of the overlying strata in the gob after the working face has been extracted^1,2^. Large coal pillars result in considerable coal resource waste^3,4^, while smaller coal pillars cause stress concentration at the pillar location due to the deformation and rotation of the over-lying strata post-mining, leading to significant surrounding rock deformation^5,6^. To overcome these challenges and eliminate the need for coal pillars, thereby improving re-source recovery rates^1,7^, this study proposes a soft mold filling wall method for gob-side entry retention. This approach constructs artificial filling walls along the roadway to enhance its overall support strength.

The design of key parameters for the gob-side filling body is crucial for ensuring the stability of roof support, a topic that has garnered extensive attention from scholars over an extended period^8–12^. Li et al.^13^ employed a gob-side filling body (with dimensions of 2.2 m in height and 1.0 m in width) to achieve a 1.1 m mining height, where the immediate roof consists of 2.45 m of mudstone and the main roof consists of 7.15 m of sandstone. Xie et al.^14^ utilized gob-side filling (with dimensions of 2.0 m in height and 1.2 m in width) to achieve a 1.6 m mining height, with the immediate roof consisting of 1.95 m of mudstone and the main roof consisting of 6.02 m of fine-grained sandstone. Tian et al.^15^ employed gob-side filling (with dimensions of 1.3 m in height and 1.0 m in width) to achieve a 1.3 m mining height, where the immediate roof is composed of 1.1 m of mudstone and the main roof consists of 3.4 m of fine sandstone. The aforementioned studies have found that, under the conditions of thin and medium-thick coal seams, a narrower gob-side filling width is sufficient to meet the load-bearing requirements^16–19^. However, with the widespread application of large-mining-height extraction techniques, the intense mining-induced stresses and the manifestation of rock pressure present significant challenges to the roadway support technology^20–27^.

Existing research and applications have predominantly focused on thin and medium-thick coal seams, where surrounding rock movement is relatively moderate. Under these conditions, the design of roadside backfill bodies did not incorporate a bearing stability coefficient, rendering such approaches inadequate for large mining height conditions. To address this gap, this study, based on the engineering context of the S1212 working face in Ningtiaota Coal Mine, employs a combined methodology of theoretical analysis, numerical simulation, and field application. A mechanical model of the surrounding rock under large mining height conditions was established, and a bearing stability coefficient for the roadside backfill body was proposed. Furthermore, a calculation formula for determining the width of the roadside backfill body under large mining height conditions was derived. Systematic numerical simulation tests were conducted under various mining heights to reveal the distribution of surrounding rock stress and the evolution law of deformation. The research outcomes were applied and validated in-field practice, aiming to provide theoretical guidance and technical support for the support design of gob-side entry retaining under similar geological and mining conditions.

## Engineering overview

**Fig. 1:**
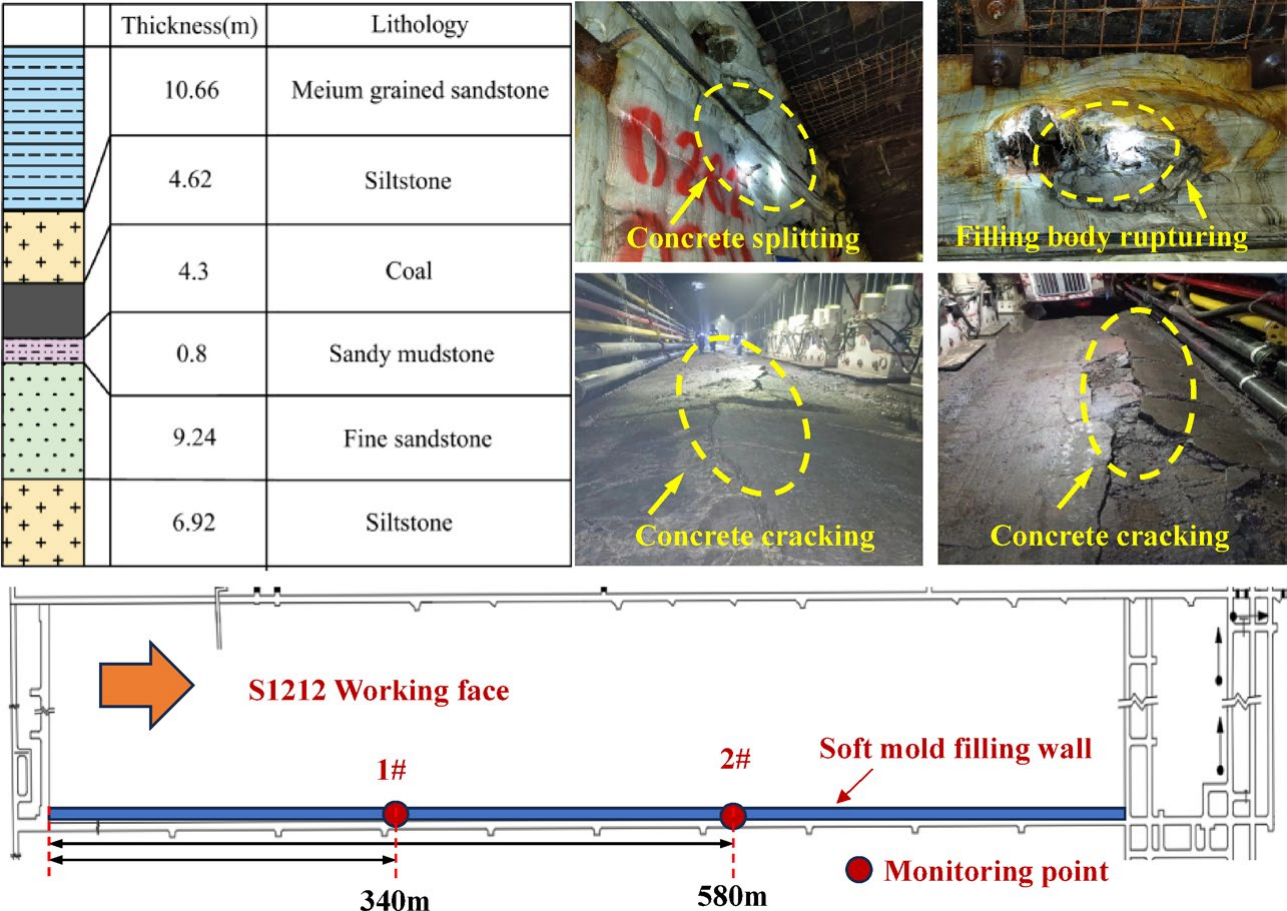
Overview of the S1212 test working face and lithological column.

The test working face S1212 is located in Shenmu County, Yulin City, Shaanxi Province. The coal seam thickness ranges from 2.6 m to 4.77 m, with an average of 4.3 m, and the burial depth varies between 164 m and 224 m. The immediate roof consists of 4.62 m of siltstone with a compressive strength of 45.3 MPa, while the main roof is composed of 10.66 m of medium-grained sandstone exhibiting a compressive strength of 40.6 MPa. Gob-side entry retaining was implemented in the belt entry of the S1212 working face, which has a strike length of 1991 m and a dip length of 344 m. The general layout and stratigraphic column of the S1212 test working face are shown in Fig. 1.

**Fig. 2:**
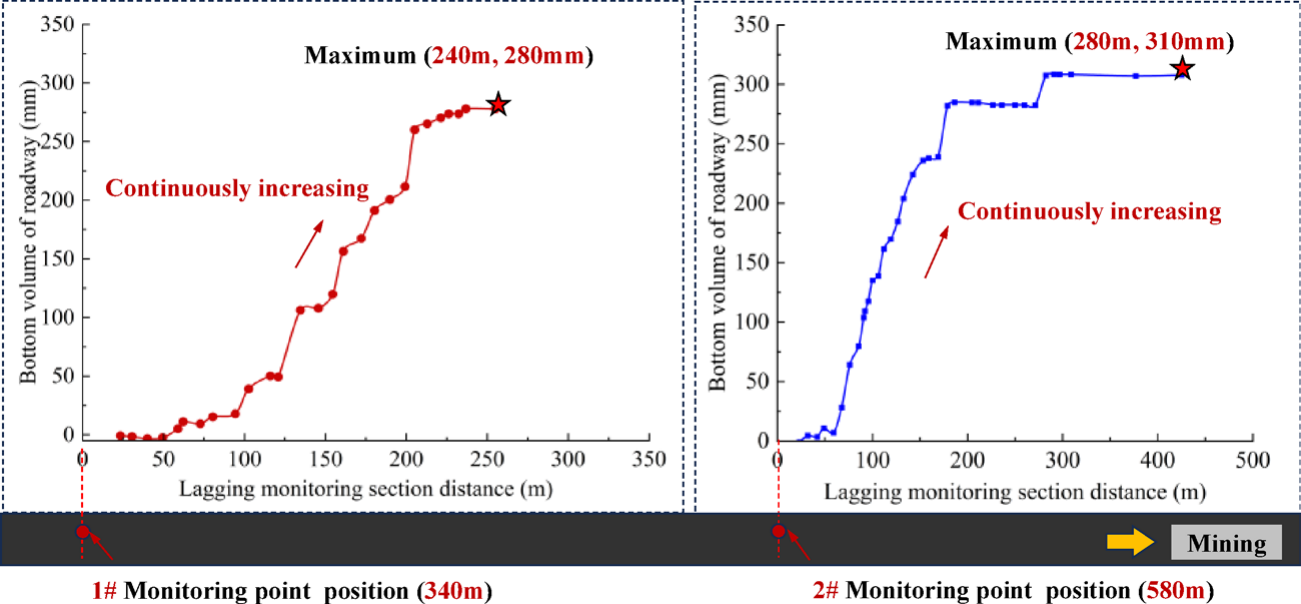
Floor heave variation curve of the roadway at the S1212 test working face.

**Fig. 3:**
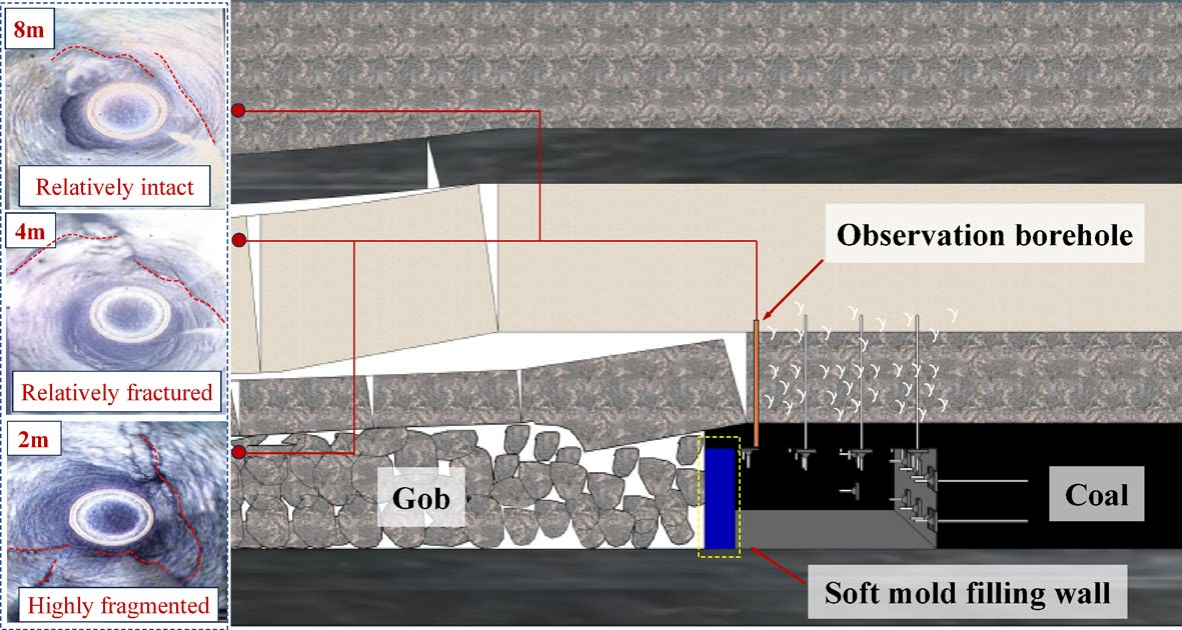
Field investigation of roof fracturing and floor deformation at the S1212.

1. Based on the geological conditions of the S1212 working face shown in Fig. 1, the high mining height leads to an extensive mining influence zone and a significant degree of stress concentration. When the overburden load and mining-induced stress are transmitted to the roadside, the limited bearing capacity of the coal seam and the roof strata, combined with the insufficient width and small effective bearing area of the roadside filling wall in its early stage, results in inadequate stress diffusion. This causes a substantial concentration of stress on the filling wall adjacent to the roadway. Simultaneously, the low early strength of the filling wall and its poor compatibility with surrounding rock deformation mean that when the concentrated stress exceeds its bearing capacity, the wall undergoes sequential failure modes: cracking, fragmentation, and bulging. This mechanism explains the observed behavior in monitoring sections #1 and #2: as the face advance distance increases and mining stress continues to accumulate, the deformation of the wall increases from 180 mm to 220 mm.
2. Based on the lithological assemblage of the S1212 working face, where both the main roof and floor consist of hard rock strata, while the immediate floor is composed of weak mudstone, the concentrated stress from the overlying hard main roof during mining is readily transmitted downward through the competent rock layers. When the stress is transferred to the mechanically weak mudstone interlayer, the limited bearing capacity of this stratum renders it a “weak zone” for stress release and deformation. This leads to bending and shear deformation of the floor. Furthermore, during the mining process, contact with water causes the floor mudstone to soften, significantly degrading its mechanical properties and drastically reducing its bearing and deformation resistance capacities. At Monitoring Point 2, the measured maximum floor heave in the roadway is 310 mm, as shown in Fig. 2.
3. Roof borehole imaging revealed that within the first 2 m from the roof surface, fractures are densely developed, and the rock mass is severely fragmented. Between 2 m and 4 m, closed-type fractures are present within the strata. Beyond 8 m, no visible fractures were observed, indicating an intact rock zone. Intensive fracturing in the shallow roof considerably diminishes the self-supporting capacity of the surrounding rock and weakens the anchoring effectiveness of roof bolts. A field image of the roof borehole inspection is shown in Fig. 3.

## Theoretical analysis

**Fig. 4:**
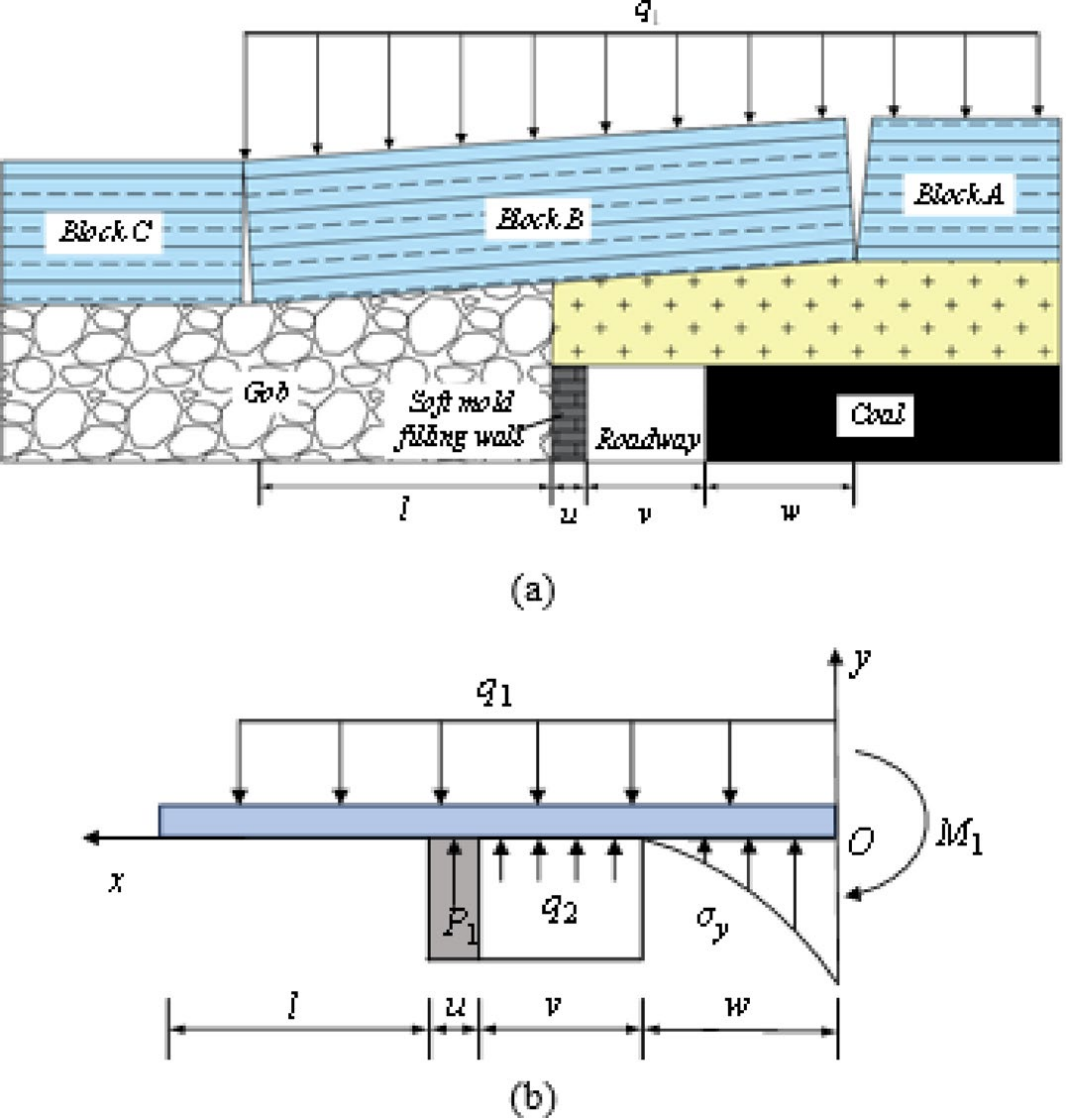
Lateral cantilever mechanical model.

After the working face is mined, the overlying strata on the gob side collapse and subside. The collapsed profile of the overburden in the dip direction is shown in Fig. 4(a). As the working face advances, delamination occurs between the immediate roof and the main roof, leading to progressive and irregular collapse and subsidence. Once the immediate roof collapses, the main roof experiences fracturing, rotation, or bending subsidence, forming a hinged structure composed of rock blocks A, B, and C.

To calculate the bearing capacity of the filling body and analyze its influencing factors, a stress analysis of block B was performed. A lateral cantilever beam mechanical model for the suspended roof was developed, as shown in Fig. 4(b). Ignoring the bending moment caused by internal stresses in block B, the roof is subjected to a uniformly distributed load *q* = *γH*, with the solid coal applying a distributed load *σ*_*y*_. The bearing capacity of the filling body and the roadway roof, denoted as *P*_1_, *q*_2_, acts at the midsection of the wall. The calculation formula for the plastic zone range u within the solid coal is given as follows:

1 $$w = \frac{{mD}}{{2\tan \varphi }}\ln \frac{{K\gamma H + \frac{{c_{0} }}{{\tan \varphi }}}}{{\frac{{c_{0} }}{{\tan \varphi }} + \frac{{p_{c} }}{D}}}$$

2 $$\sigma _{y} = \left( {\frac{{c_{0} }}{{\tan \varphi }} + \frac{{p_{c} }}{D}} \right)\mathrm{e} ^{{\left( {\frac{{2x\tan \varphi }}{{{\mathrm{m}}D}}} \right)}} - \frac{{c_{0} }}{{\tan \varphi }}$$

Where: *φ* represents the internal friction angle of the rock interface; *D* represents the lateral pressure coefficient; *γ* represents the average unit weight of the overlying strata (kN/m³); *K* represents the stress concentration factor; *H* represents the working face burial depth (m); *m* represents the mining height of the coal seam (m); *c*_*0*_ represents the cohesion at the interface between the coal seam and the roof/floor strata (Pa).

Taking block B as a whole, the mechanical equilibrium method is employed to establish the equilibrium equations.

$$\Sigma F_{y} = 0$$:

3 $$\int_{0}^{w} {\sigma _{y} dx} + P_{1} + q_{2} v = q_{1} L$$

$$\Sigma M_{c} = 0$$:

4 $$M_{1} + \int_{0}^{w} {\sigma _{y} xdx} + P_{1} \left( {v + w + \frac{u}{2}} \right) + q_{2} v\left( {w + \frac{v}{2}} \right) = \frac{1}{2}q_{1} L^{2}$$

5 $$L = u + v + w + l$$

Assuming the lateral ultimate bending moment of the roof is $M_{1} \le M_{u}$, it satisfies the following relationship:

6 $$\begin{gathered} P_{1} \ge k_{1} \left( {\frac{2}{{v + u}}\left\{ {\frac{1}{2}q_{1} L^{2} - M_{u} - \left( {\frac{{mD}}{{2\tan \varphi }}} \right)^{2} \left[ {\left( {K\gamma H + \frac{{c_{0} }}{{\tan \varphi }}} \right)\zeta + \left( {\frac{{P_{c} }}{D} - K\gamma H} \right) - \frac{{c_{0} }}{{2\tan \varphi }}\zeta ^{2} } \right]} \right.} \right. \hfill \\ {\text{ }}\left. {{\text{ - }}q_{1} L\left( {\frac{{mD}}{{2\tan \varphi }}\zeta + \frac{v}{2}} \right) + \left[ {\frac{{mD}}{{2\tan \varphi }}\left( {K\gamma H - \frac{{P_{c} }}{D}} \right) - \frac{{c_{0} mD}}{{2\tan ^{2} \varphi }}\zeta } \right]\left. {\left( {\frac{{mD}}{{2\tan \varphi }} + \frac{v}{2}} \right)} \right\}} \right) \hfill \\ \end{gathered}$$

7 $$\zeta = \ln \frac{{K\gamma H + \frac{{{\mathrm{c}}_{{\mathrm{0}}} }}{{\tan \varphi }}}}{A}$$

**Fig. 5:**
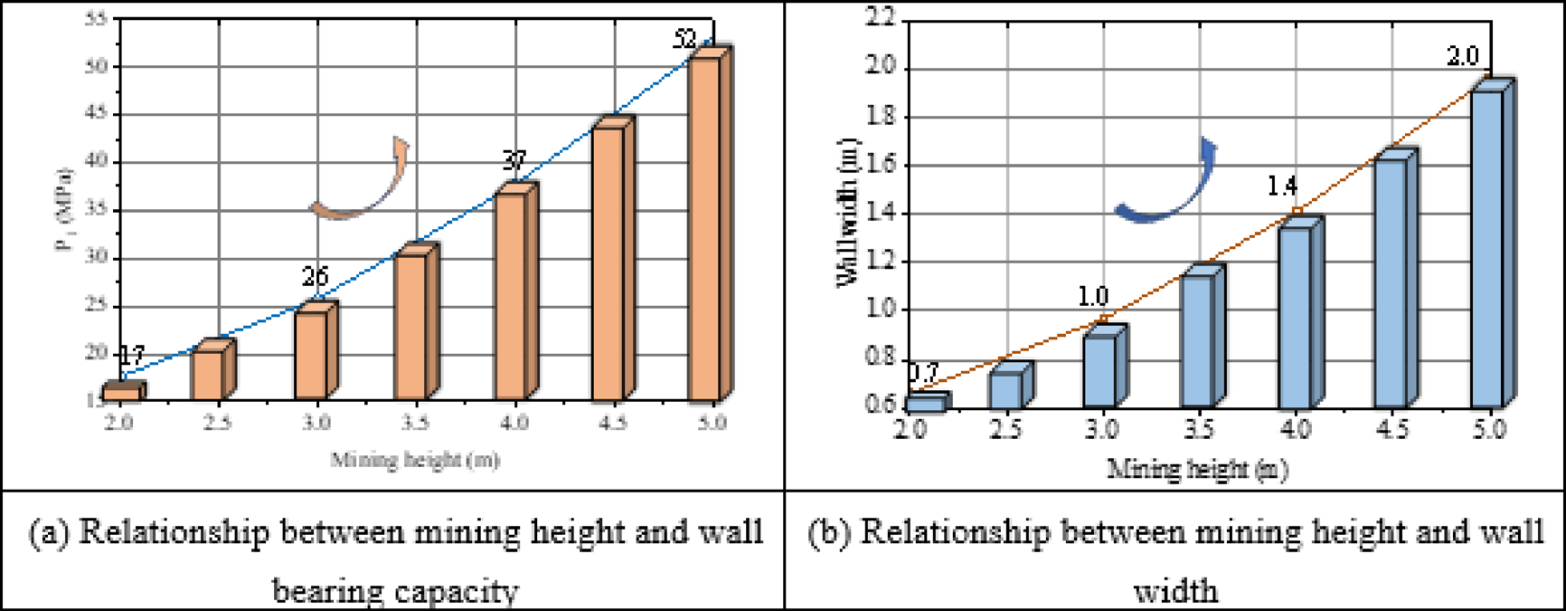
Curve of variation under different mining heights.

To investigate the influence of various factors on the wall’s bearing capacity, the coal seam mining height is considered within the range *m* = 2–5 m, following previous studies^28,29^. Based on the geological conditions, the parameters are set as: *q*_1_ = 4.85 MPa, *H* = 194 m, *K* = 1, *D* = 0.5, *v* = 6 m, *u* = 1.2 m, *w* = 4.71 m, and *l* = 18.9 m. The roadway support strength is set as *p*_c_ = 0.3 MPa. The cohesion at the coal seam–roof/floor interface is *c*_0_ = 0.6 MPa, while the corresponding internal friction angle is *φ* = 30°. Considering a stability coefficient of k₁ = 1.5, and with the field concrete strength being C40, the bearing capacity of the soft mold wall under different mining heights and the corresponding design values of wall width were calculated using Eq. (6), the displacement variations under different parameter conditions are obtained, as shown in Fig. 5.

From Eq. (6), it can be observed that as the mining height of the working face increases, the required lateral support resistance along the gob-side entry also increases. Therefore, for working faces with large mining heights, the required bearing capacity of the entry support can be achieved by either increasing the width of the filling wall or enhancing the strength of the filling material.

### Numerical simulation

To determine the optimal width of the filling wall along the roadway at different mining heights, this study focuses on the S1212 working face at Ningtiaota Coal Mine. Numerical simulations of gob-side entry retaining were conducted across different mining heights and soft mold filling wall widths. The stress distribution and deformation progression of the surrounding rock under these conditions were systematically analyzed.

### Model establishment and scheme design

#### Monitoring scheme

**Fig. 6:**
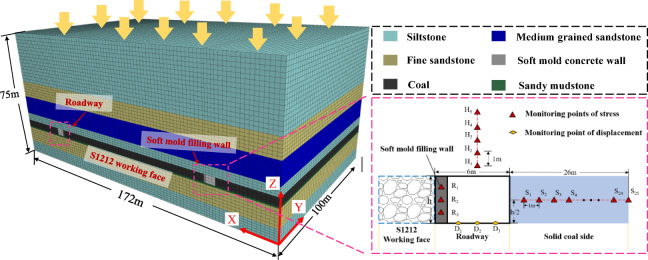
Numerical model and monitoring layout.

A model was developed to reflect the geological and engineering conditions of the S1212 working face at Ningtiaota Coal Mine. The model dimensions are 172 m × 100 m × 75 m, with a simulated working face length of 100 m, a coal seam thickness of 4 m, and roadway dimensions of 6 m × 4 m. The average unit weight of the overburden strata in the simulated area is 24.5 kN/m³. Based on the principle that the vertical in-situ stress equals the product of the average unit weight of the overburden strata and the coal seam burial depth, the vertical in-situ stress was calculated to be 3.14 MPa. Assuming the rock layer is a continuous homogeneous medium, ignoring the randomly distributed joints and fissures inside. The soft mold filling wall next to the roadway reaches the design strength at once, and the strength growth during the curing period is not considered for the time being. The main focus is on analyzing the vertical stress variation law inside the flexible formwork wall. Accordingly, a uniformly distributed load of 3.14 MPa was applied to the top of the model. The simulation model and monitoring layout are presented in Fig. 6.

Three vertical stress monitoring points, spaced 1 m apart, were positioned at the mid-thickness of the soft mold concrete wall. Three displacement monitoring points, spaced 1 m apart, were installed on the roadway floor to monitor floor heave deformation throughout the mining process. Additionally, monitoring points were installed at 1 m intervals along the solid coal rib, totaling 25 points. The rock’s physical and mechanical parameters summarized in Table 1.

**Table 1:** Physical and mechanical parameters of rock.

Lithology	Bulk modulus (GPa)	Shear modulus (GPa)	Tensile strength (MPa)	Internal friction angle (°)
Medium-grained sandstone	5.46	4.27	0.58	41.33
Siltstone	3.54	7.39	0.86	38.29
Mudstone	4.40	3.30	0.55	33.00
Fine Sandstone	7.02	4.63	0.64	40.91
Coal	3.95	4.63	0.64	39.69

#### Test scheme

To define the optimal width of the filling body along the roadway under varying mining heights, four experimental schemes I_*i*_ – IV_*i*_ were designed based on actual site conditions. These correspond to mining heights of 2 m, 3 m, 4, m and 5 m, respectively. The subscript iii denotes the corresponding widths of the soft mold concrete wall, which are 1.0 m, 1.2 m, 1.4 m, 1.6 m, 1.8 m, and 2.0 m. The specific experimental schemes are detailed in Table 2.

**Table 2:** Width of soft mold filling wall under different mining heights.

Schemes	Mining height (m)	Width of soft mold filling wall (m)
I_*i*_	2	1.0、1.2、1.4 1.6、1.8、2.0
II_*i*_	3	
III_*i*_	4	
IV_*i*_	5	

### Orthogonal experimental design

In order to analyze the influence of different factors on the stability of the surrounding rock in gob-side entry retaining, an orthogonal experimental design was conducted. Three key controllable factors were selected: the strength of the filling wall, the density of roof support, and the strength of the floor. A three-factor orthogonal test was conducted with each factor at three levels.

The compressive strength of the filling wall typically ranges from 30 to 50 MPa, and under strong dynamic pressure conditions, it must exceed 40 MPa^30,31^. The roof support density can be adjusted by modifying the spacing of bolts and cables, with a typical densification range of 20% to 50%^32^. The floor rock is classified into three categories based on strength: weak, medium-hard, and hard, corresponding to tensile strengths of < 1.5 MPa, 1.5–2.5 MPa, and > 2.5 MPa, respectively^33,34^.

**Table 3:** Three-factor three-level experimental design.

Factor	Filling wall strength	Support density	Floor strength
Level 1	30 MPa	K = 1.0	1 MPa
Level 2	40 MPa	K = 1.25	2 MPa
Level 3	50 MPa	K = 1.50	3 MPa

Considering the specific geological conditions of the Ningtiaota S1212 working face, which include a burial depth ranging from 166.6 m to 212.3 m, a siltstone immediate roof, and a floor primarily composed of mudstone and fine-grained sandstone, this study established three factors for experimental design, each at three levels. The compressive strength of the filling wall was set at 30, 40, and 50 MPa, corresponding to its specified C40 strength grade. The support density was based on the original design as the baseline (K = 1.0), with two enhanced levels representing 25% (K = 1.25) and 50% (K = 1.50) increases. The floor strength was assigned three representative levels, namely weak (1 MPa), medium hard (2 MPa) and hard (3 MPa), based on exploration reports and uniaxial tensile strength tests conducted on borehole cores.

**Fig. 7:**
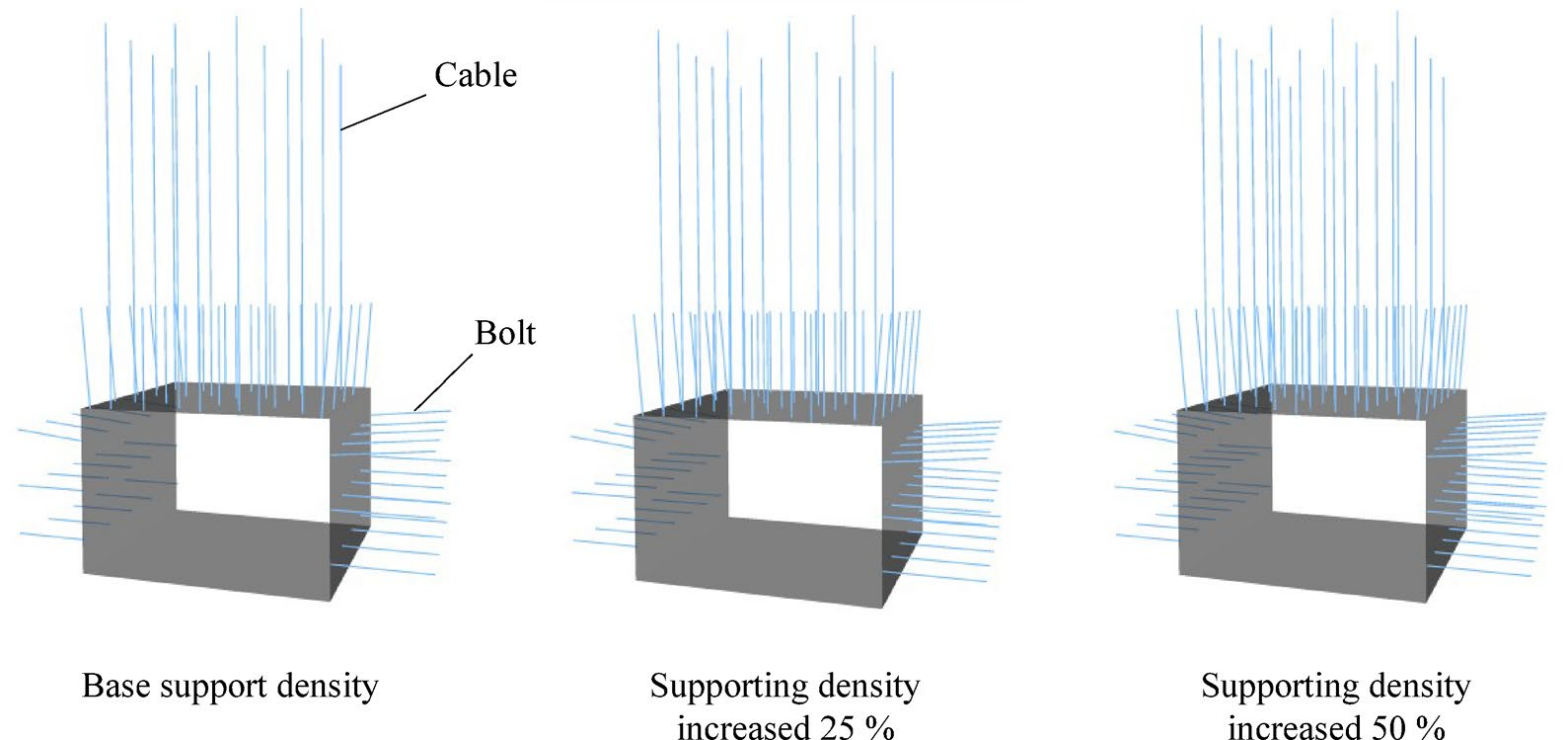
Orthogonal test simulation conditions (taking different support densities as an example).

According to the three-factor, three-level orthogonal experimental design presented in Table 3, nine simulation conditions (working conditions) were established. Representative configurations of the orthogonal test simulations are illustrated in Fig. 7, with the values of the relevant influencing factors assigned in accordance with Table 4. Taking the deformation of the surrounding rock as the evaluation index, a numerical simulation analysis scheme was designed and carried out for each condition. The simulation conditions and corresponding results are summarized. By conducting a range analysis on the surrounding-rock deformation obtained under each condition, the relative significance of the key parameters in gob-side entry retaining on the stability of the surrounding rock was determined. The findings provide a direct basis for optimizing subsequent construction parameters.

**Table 4:** Orthogonal test conditions and results.

Operating condition number	Filling wall strength /MPa	Support density/K	Floor tensile strength /MPa	Deformation of surrounding rock /mm
1	30	1.0	1	86.2
2	30	1.25	2	68.5
3	30	1.50	3	55.0
4	40	1.0	2	52.3
5	40	1.25	3	42.1
6	40	1.50	1	65.8
7	50	1.0	3	34.7
8	50	1.25	1	49.5
9	50	1.50	2	39.6

### Analysis of orthogonal experimental results

The range (Z) analysis method was employed to assess the significance of key gob-side entry retaining parameters on the deformation of the surrounding rock. The range is calculated using the following formula:

8 $$Z = \max (K_{i} ) - \min (K_{i} )$$

In Eq. (8), *X*_i_ is the sum of the test values at the same level in this column, *K*_i_ is the average of *X*_i_; Z is the range^35^.

According to the numerical simulation results of the orthogonal test in Table 5, the range (Z) of surrounding rock deformation can be calculated using Eq. (8), as shown in Table 5.

**Table 5:** Range of deformation in tunnel surrounding rock.

Indicator	Filling wall strength	Support density	Floor strength
X_1_	209.7 mm	173.2 mm	201.5 mm
X_2_	160.2 mm	160.1 mm	160.4 mm
X_3_	123.8 mm	160.4 mm	131.8 mm
K_1_	69.90 mm	57.73 mm	67.17 mm
K_2_	53.40 mm	53.17 mm	53.47 mm
K_3_	41.27 mm	53.07 mm	43.93 mm
Range Z	28.63 mm	4.66 mm	23.24 mm

According to Table 3, the deformation of the surrounding rock exhibits a clear correlation with the key influencing factors considered in the gob-side entry retaining scenario. A comparison of the ranges across different parameter levels shows: filling wall strength (Z = 28.63 mm) > floor strength (Z = 23.24 mm) > support density (Z = 4.66 mm). This indicates that the sensitivity of surrounding rock deformation to these factors follows the order: filling wall strength exerts the most significant influence, followed by floor strength, with support density having the least effect.

**Fig. 8:**
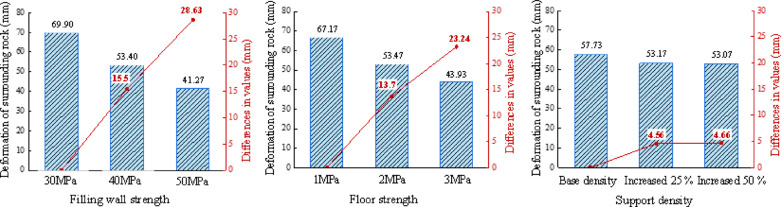
Trend chart of roadway surrounding rock deformation under different influencing factors.

To visually demonstrate the influence of key gob-side entry retaining parameters, trend graphs were plotted based on the orthogonal test results, illustrating the effects of the filling wall strength, support density, and floor strength on surrounding rock deformation at each factor level, as presented in Fig. 8.

This trend chart clearly reveals the influence patterns and relative importance of the three factors on surrounding rock deformation. The filling wall strength is the most sensitive controlling factor, with its increase from 30 MPa to 50 MPa significantly reducing the range Z by 28.63 mm. The floor strength, as a key geological condition, leads to a reduction in the range Z of 23.24 mm when transitioning from weak to hard rock. In contrast, the influence of support density is relatively limited: a 50% increase in density only lowers the maximum deformation by 4.66 mm. Based on the range analysis, the significance of factors affecting surrounding rock deformation can be ranked as: filling wall strength > floor strength > support density. These results indicate that in gob-side entry retaining projects, priority should be given to ensuring high filling wall strength and implementing appropriate floor treatment, with roof support maintained at a reasonable level without excessive reinforcement.

### Numerical results analysis

Numerical simulations were conducted to investigate the effects of mining height and soft mold filling wall width on the wall’s vertical stress, stress within the solid coal rib, and roadway floor heave. The main findings are summarized as follows:

#### Stress analysis of soft mold filling wall

**Fig. 9:**
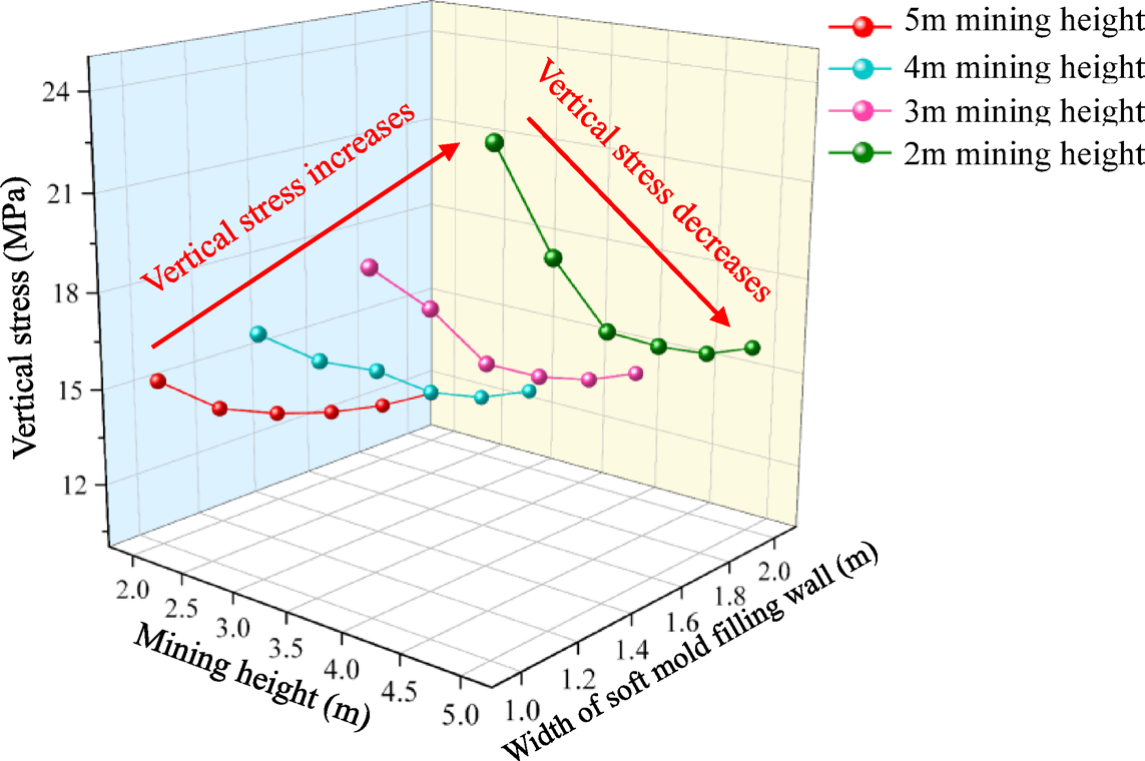
Stress variation curve of soft mold filling wall.

By comparing the variation patterns of vertical stress within the soft mold filling wall under different mining heights, it is observed that, when mining height remains constant, the vertical stress within the wall decreases as the wall width increases, as illustrated in Fig. 9. For example, at a mining height of 2 m, the vertical stress within the wall decreases by a factor of 1.3 as the wall width increases from 1.0 m to 1.4 m, and decreases by a factor of 1.1 as the width increases from 1.4 m to 2.0 m. This suggests that increasing the wall width enhances its load-bearing capacity and redirects the overburden pressure towards the solid coal rib, thereby mitigating vertical stress concentration within the wall. However, beyond a certain wall width, further increases yield diminishing reductions in vertical stress.

When the width of the soft mold wall remains constant, an increase in height results in a rise in wall’s vertical stress. For instance, at a wall width of 1.2 m, the vertical stress increases by a factor of 1.7 as the height rises from 2 m to 5 m; at a width of 1.4 m, it increases by a factor of 1.6 over the same range. This indicates that vertical stress concentration within the wall is more strongly influenced by mining height. For gob-side entry retaining under large mining height conditions, increasing the wall width is essential to enhance the wall’s load-bearing capacity.

#### Stress analysis of solid coal

**Fig. 10:**
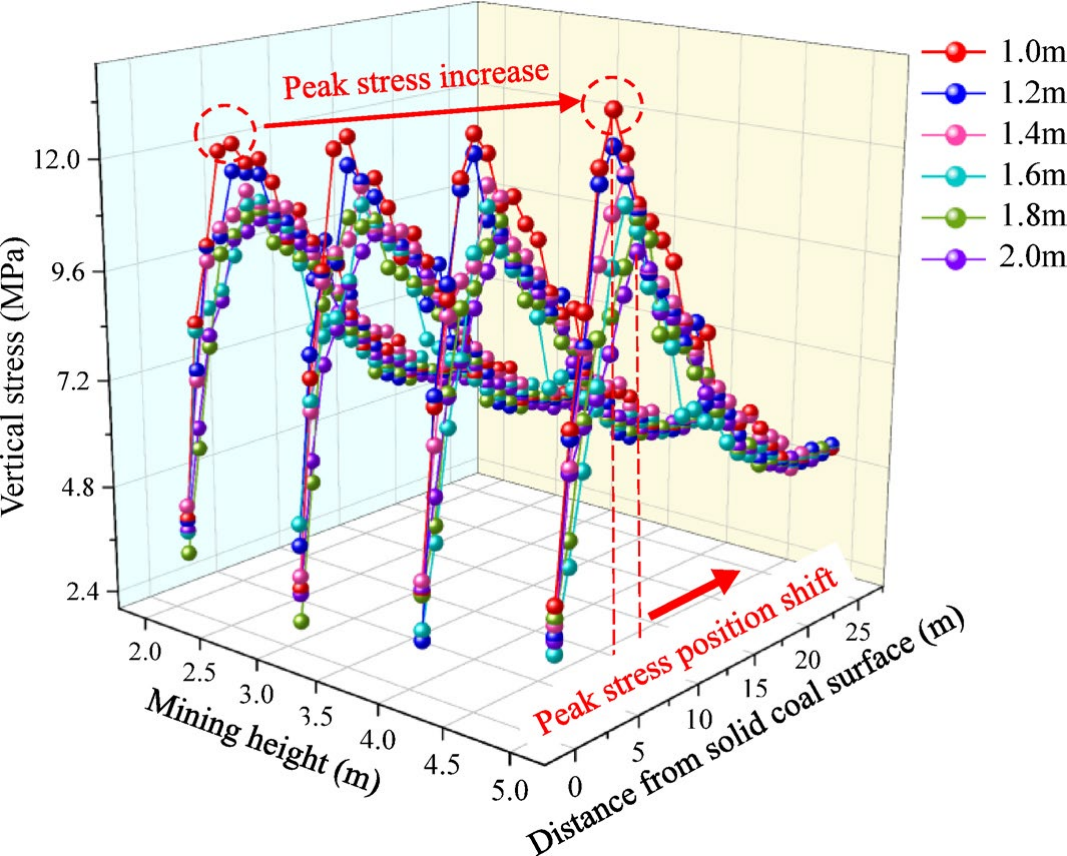
Stress variation curve of the solid coal.

The analysis of stress variation in the solid coal shows that, with the mining height kept constant, the peak stress decreases and its location shifts deeper into the coal rib as the width of the soft mold filling wall increases, as illustrated in Fig. 10. For instance, at a mining height of 2 m, the peak stress decreases by a factor of 1.2 and shifts 1.1 m deeper into the coal rib as the wall width increases from 1 m to 2.0 m. This suggests that a wider filling wall creates a larger synergistic load-bearing zone with the surrounding rock, effectively sharing the overburden load and shifting the peak stress deeper into the coal rib. However, as the width of the soft mold filling wall remains constant, increasing the mining height results in a rise in peak stress on the side of the solid coal rib, with the peak location shifting closer to the coal rib surface.

#### Deformation of surrounding rock in roadway

**Fig. 11:**
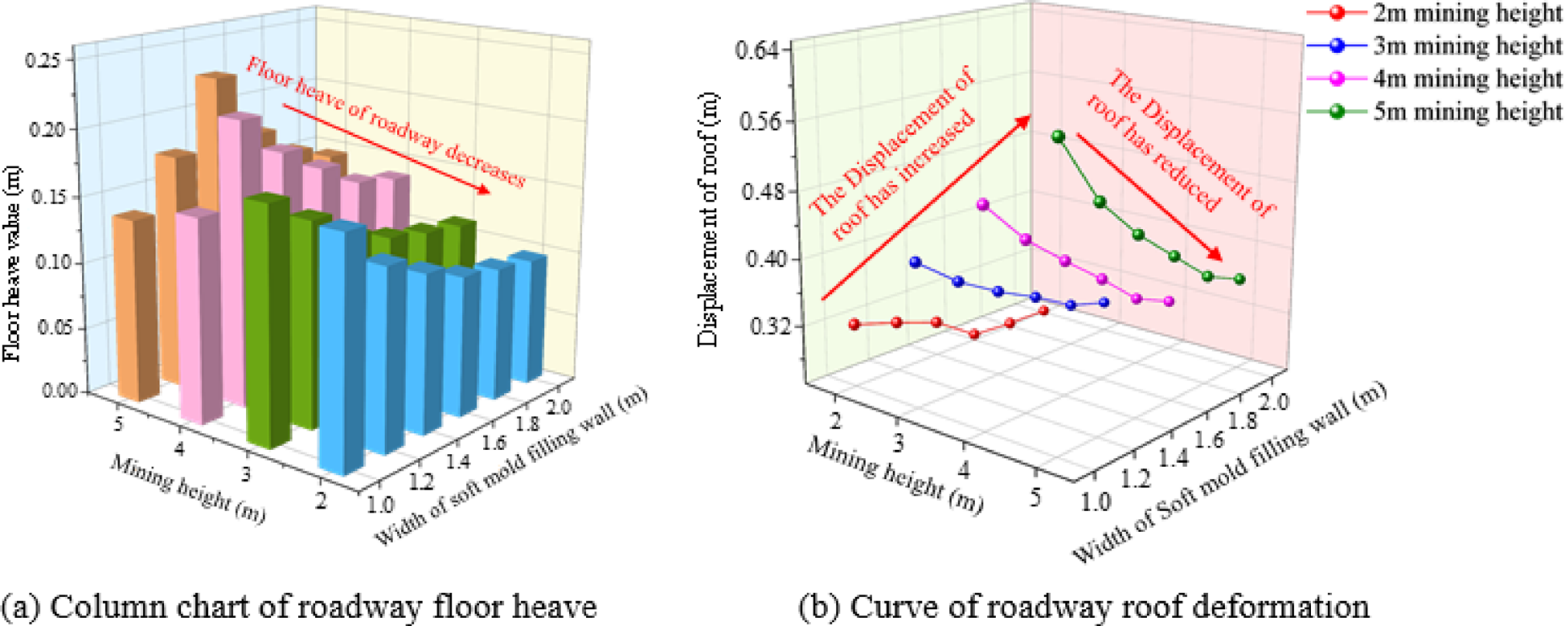
Surrounding rock deformation patterns at different mining heights.

Analysis of the floor heave variation reveals that, when mining height remains constant, floor heave gradually decreases as the width of the soft mold wall increases. However, when the width of the soft mold filling wall remains constant, increasing the mining height of the working face results in an increase in roadway floor heave. The variation curve of the floor heave is shown in Fig. 11 (a). Roof deformation analysis indicates that roadway subsidence intensifies with increasing mining height. As the mining-induced stress redistributes, larger mining heights significantly weaken the bearing capacity of the roof strata and enlarge the range of plastic failure, leading to greater roof deformation. The curve of roadway roof deformation is shown in Fig. 11 (b).

**Fig. 12:**
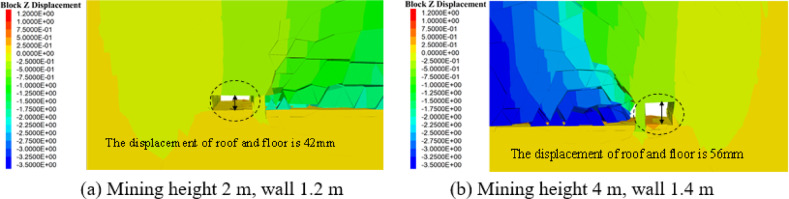
Deformation contour map of the roadway surrounding rock.

Numerical simulations indicate that, at a mining height of 2 m, the roadway floor heave decreases with increasing width of the soft mold filling wall. For instance, when the mining height is 2.0 m with a wall width of 1.2 m, the roadway floor heave reaches 176 mm. In contrast, at a mining height of 4.0 m with a wall width of 1.4 m, the floor heave increases to 246 mm. Representative deformation cloud maps of the surrounding rock under these conditions are shown in Fig. 12(a) and (b).

## Field application

### Engineering recommendations

Based on numerical simulation experiments conducted under varying mining heights and soft mold filling wall widths, the effects of these parameters on the wall’s vertical stress, solid coal rib stress, and roadway floor heave were analyzed. The following engineering recommendations are proposed in consideration of actual site conditions:

1. Effect of mining height: As mining height increases from 2 m to 5 m, both the vertical stress on the wall and the vertical stress within solid coal rib significantly increase, accompanied by greater roadway floor heave. Therefore, in practical mining operations, adopting stratified mining methods to appropriately reduce mining height is recommended to maintain roadway surrounding rock stability.
2. Effect of wall width: With increasing soft mold filling wall width, both the vertical stress within the wall and the roadway floor heave decrease. However, when the wall width exceeds a certain threshold under a constant mining height, the marginal improvement in floor heave control diminishes. Therefore, considering actual field conditions, a filling wall width of no less than 1.4 m is recommended when the height ranges from 2 m to 4 m.
3. On-site construction recommendations: A comprehensive monitoring system should be established to track surrounding rock deformation, wall stress, and the stress on roadway support structures. In particular, under large mining height conditions, real-time monitoring and assessment of the stress and deformation of the gob-side filling wall should be conducted, with dynamic optimization of design parameters for entry retaining using the monitoring results.

### Analysis of monitoring results

**Fig. 13:**
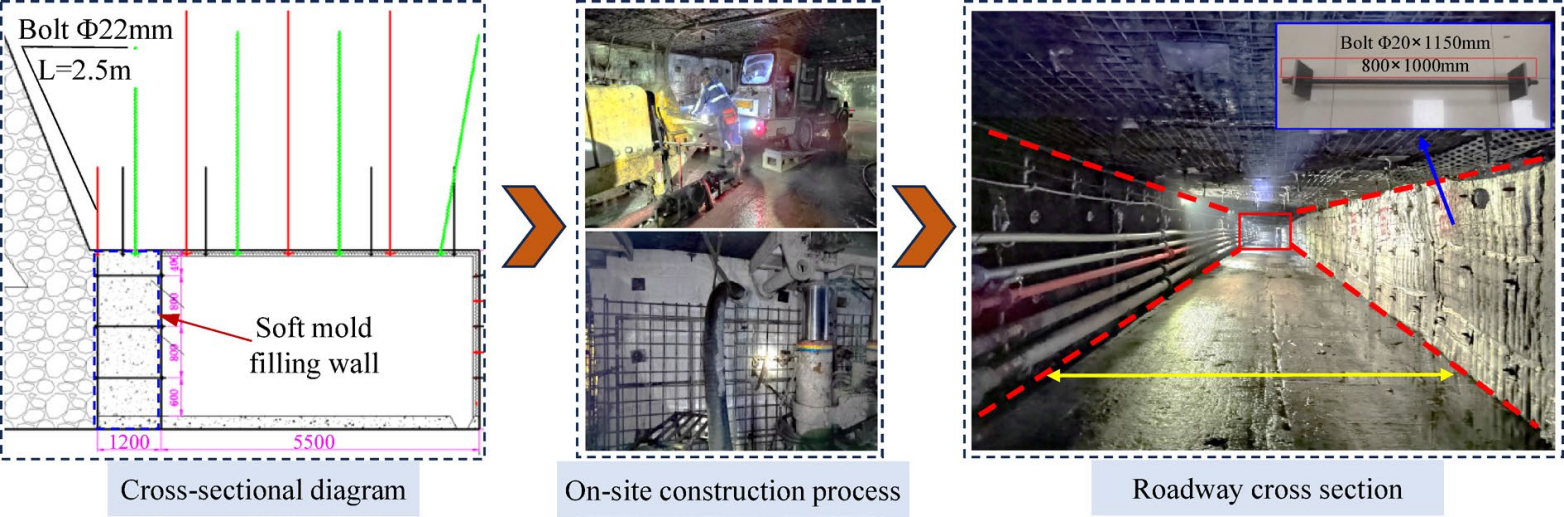
The cross section of gob-side entry retaining.

**Fig. 14:**
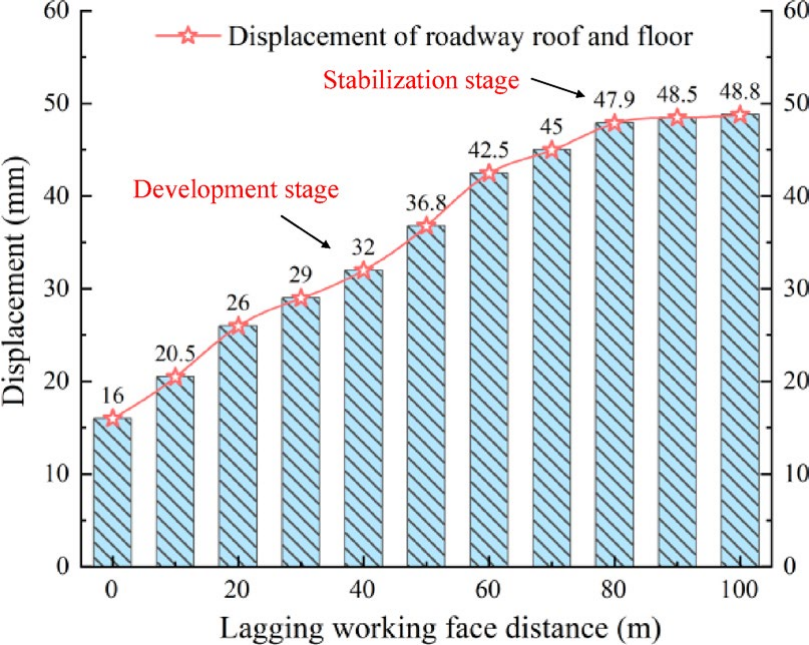
The curve of surrounding rock deformation.

**Fig. 15:**
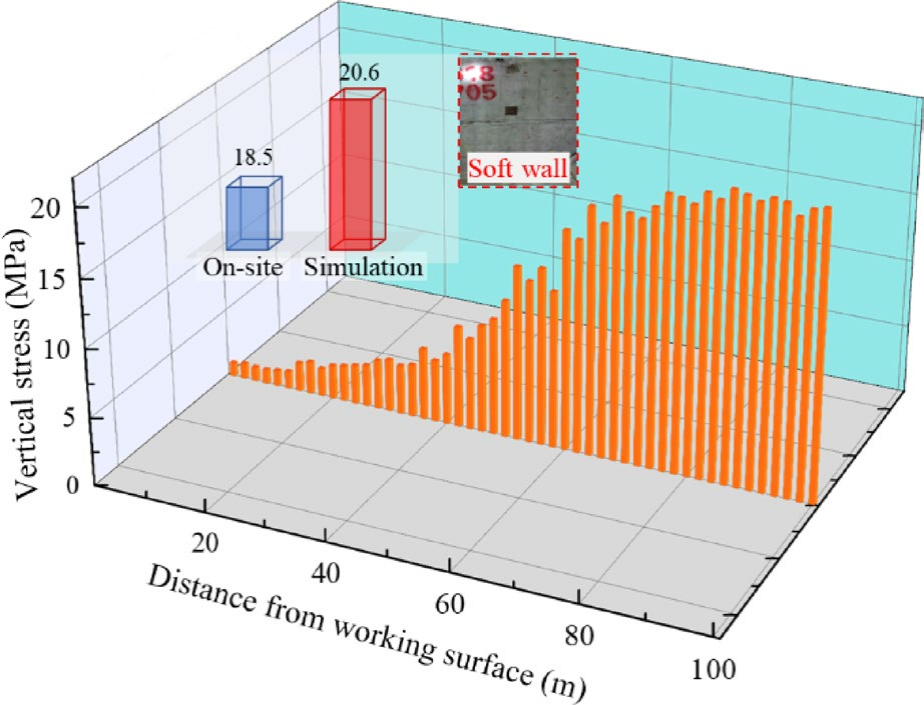
The curve of soft mold concrete wall stress.

Building on the results of the numerical simulation and considering construction efficiency, a field test and practical application were conducted in the belt conveyor roadway. A soft mold filling wall with a width of 1.2 m was constructed on the gob side. The filling structure was reinforced using tie rods (Φ20 × 1150 mm) with a spacing of 800 × 1000 mm. Roof reinforcement was achieved through anchor cables (Φ21.8 × 7000 mm), arranged at 1500 × 2400 mm intervals. The results of the on-site implementation are shown in Fig. 13. To evaluate the effectiveness of gob-side entry retention in practical applications, monitoring cross-sections were installed on-site to examine the deformation of the surrounding rock, the stress response of bolts and anchor cables, and the stress distribution within the soft mold concrete wall. The monitoring results are shown in Figs. 14 and 15.

1. Surrounding rock deformation: Regarding surrounding rock deformation, the monitoring curves exhibit a trend of initial increase followed by stabilization, which can be categorized into a deformation growth zone and a deformation stabilization zone. The surrounding rock deformation gradually stabilized at a location 80 m behind the working face, with a roof-to-floor convergence measured at 48.8 mm, as shown in Fig. 14. The maximum roof-to-floor convergence obtained from the numerical simulation was 56 mm, resulting in a discrepancy rate of 12.9%.
2. Stress in the soft mold filling wall: The stress in the soft mold concrete wall stabilized at 18.5 MPa as the working face was 70 m behind, as shown in Fig. 15. Numerical simulations for walls of the corresponding width yielded a similar stabilized stress of 20.6 MPa, with a deviation of 11.3% compared to field measurements. The on-site measured average vertical stress of the filling wall is 11.8 MPa, while the corresponding numerical simulation yields an average value of 13.2 MPa, resulting in an average deviation rate of 11.8%.
3. The optimized scheme with a wall width of 1.2 mm not only ensures roadway stability but also exhibits favorable overall performance in terms of comprehensive engineering benefits. From an economic standpoint, while the increased wall width raises the cost of filling materials, this solution effectively offsets the potentially high expenses associated with repairing and reconstructing unstable narrow walls. Additionally, the successful implementation of gob-side entry retention technology has significantly increased the coal recovery rate, generating considerable additional production revenue and thus delivering notable overall economic benefits. From an operational perspective, the optimized support system substantially prolongs the stable service period of the wall, greatly reduces the frequency and duration of roadway maintenance, ensures continuous and stable roadway operation, and effectively avoids production losses due to maintenance interruptions. The mechanized soft mold filling technology offers high operational efficiency, significantly boosting construction productivity. It is well-suited to the rapid-advancement requirements of high mining height faces and provides robust support for coordinating mining and production activities.

The vertical stress of the wall, whether measured on-site or obtained through numerical simulation, remains below the standard compressive strength of C40 concrete. On-site deformation monitoring in the roadway further revealed that the maximum vertical deformation of the wall was 48.8 mm, with no observed signs of instability such as crack propagation or local collapse. In light of the identified stress discrepancy, the following measures are proposed to enhance wall stability: (a) optimizing the mix proportion of soft mold concrete to improve early-strength uniformity. (b) adjusting the pretension force of anchor bolts to mitigate stress concentrations in the surrounding rock, and (c) introducing real-time stress monitoring during construction to dynamically adjust support timing, thereby ensuring the wall’s long-term stable bearing performance.

## Conclusions

1. A mechanical model of the surrounding rock structure for roadside backfilling was established, and a bearing stability coefficient for the roadside backfill body under large mining height conditions was proposed. Based on this, a quantitative relationship expression between mining height and the support width of the soft mold filling wall was derived, and curves illustrating the corresponding filling wall widths for different mining heights were plotted. For a mining height of 4 m, the width of the soft mold filling wall is recommended to be no less than 1.4 m.
2. At a constant mining height, as the width of the soft mode wall increases, the vertical stress within the wall gradually decreases, and the peak stress in the solid coal side decreases with its location shifting deeper into the coal mass. Conversely, with a fixed wall width, an increase in mining height elevates the vertical stress in the wall. For instance, when the mining height increases from 2 m to 5 m, the vertical stress in the wall increases by 1.7 times.
3. These engineering recommendations, derived from theoretical and numerical analysis, were validated in a field trial at the S1212 working face using the soft mold filling method for gob-side entry retaining. The results show that the deformation of surrounding rock tends to be stable within 80 m behind the working face, and the convergence of roof and floor is 48 mm, which realizes the effective control of roadway stability.

## Data Availability

The datasets used and analysed during the current study available from the corresponding author on reasonable request.
